# Usefulness of the APTT waveform for the diagnosis of DIC and prediction of the outcome or bleeding risk

**DOI:** 10.1186/s12959-019-0201-0

**Published:** 2019-06-28

**Authors:** Kei Suzuki, Hideo Wada, Takeshi Matsumoto, Makoto Ikejiri, Kohshi Ohishi, Yoshiki Yamashita, Hiroshi Imai, Toshiaki Iba, Naoyuki Katayama

**Affiliations:** 1 Emergency Critical Care Center, Mie University Graduate School of Medicine, Tsu, Mie Japan; 2 Departments of Molecular and Laboratory Medicine, Mie University Graduate School of Medicine, Tsu, Mie 514-8507 Japan; 3 Division of Blood Transfusion Medicine and Cell Therapy, Mie University Graduate School of Medicine, Tsu, Mie Japan; 4 Central laboratory, Mie University Graduate School of Medicine, Tsu, Mie Japan; 5 Department of Hematology and Oncology, Mie University Graduate School of Medicine, Tsu, Mie Japan; 6 Department of Emergency and Disaster Medicine, Juntendo University Graduate School of Medicine, Tokyo, Japan

**Keywords:** APTT waveform, Hypofibrinogenemia, Bleeding, Outcome

## Abstract

**Background:**

The usefulness of the activated partial thromboplastin time (APTT) waveform has been reported in hemophilia, acquired hemophilia and monitoring for anticoagulants.

**Material and methods:**

The APTT waveform was examined in patients suspected of having disseminated intravascular coagulation (DIC) to analyze its usefulness for the diagnosis of DIC or the prediction of the outcome or bleeding risk.

**Results:**

DIC with fibrinogen < 2 g/L was frequently associated with infectious diseases (43.3%). The heights of the first derivative peak (1stDP) and second DP (2ndDP) were extremely low in DIC, especially DIC with hypofibrinogenemia, but high in infectious patients without DIC. The peak time and width of the 1stDP and 2ndDP were prolonged in patients with DIC. The heights of the 1^st^DP and 2^nd^DP were markedly low in patients with a poor outcome or those with hemoglobin < 8.0 g/dl.

**Discussion and conclusion:**

As bleeding type DIC was observed in infectious DIC, DIC without hypofibrinogenemia might switch to DIC with hypofibrinogenemia by the progression of DIC. The height of the 1^st^DP and 2^nd^DP is useful for the diagnosis of DIC and prediction of the bleeding risk or outcome.

## Introduction

Disseminated intravascular coagulation (DIC) [1, 2] is a serious and fatal disease that causes microvascular thrombosis associated with thrombocytopenia, a bleeding tendency with hyperfibrinolysis and organ failure. The basic mechanism underlying the onset for DIC is the marked activation and consumption of the coagulation system followed by the activation of secondary fibrinolysis [3]. DIC also has several clinical subtypes, including asymptomatic type, marked bleeding type, organ failure type and complication types such as thrombotic microangiopathy (TMA) [4, 5]. Marked bleeding type DIC is associated with hypofibrinogenemia and is generally observed in patients with leukemia, trauma or aneurysm, while organ failure type DIC is associated with an elevated fibrinogen level and generally observed in infectious DIC such as sepsis [5, 6]. Asymptomatic DIC is considered pre-DIC.

The activated partial thromboplastin time (APTT) was previously considered to be an end-point clotting time assay which was useful for diagnosing an intrinsic pathway of coagulation factor deficiency as hemophilia and acquired hemophilia [7, 8], antiphospholipid antibody (aPL), such as lupus anticoagulant (LA) [9], or for monitoring unfractionated heparin (UFH) treatment [10]. Optical coagulation analyzers can visualize the clotting curve, and abnormal biphasic clotting curves have been reported to be associated with DIC [11, 12].

The ACL TOP analyzer for the using APTT-synthetic phospholipids (SPs) recently showed the associated first- and second-derivative peaks (1^st^ and 2^nd^ DPs, respectively) [13] It has been reported that the evaluation of the 1^st^ and 2^nd^ DPs in the APTT is valuable for diagnosing coagulation factor abnormalities and monitoring anti-Xa inhibitor [14, 15].

In this study, we measured and analyzed the APTT waveform in patients suspected of having DIC and examined the relationship between the bleeding risk or outcome and the parameters of the APTT waveform.

## Materials and methods

### Patients

A total of 211 patients suspected of having DIC, who had hemostatic abnormalities such as platelet count < 120,000/μl, fibrinogen and fibrin degradation products (FDP) > 10.0 μg/ml, prothrombin time (PT) ratio > 1.27 or fibrinogen < 150 mg/dl, at Mie University Hospital from June 1, 2011 to December 31, 2017, were enrolled in the study. DIC was diagnosed according to the Japanese Ministry of Health, Labor and Welfare (JMHLW) diagnostic criteria for DIC [16]. A JMHLW DIC score of < 6 points, 6 points or ≧7 points was defined as non-DIC, pre-DIC and DIC, respectively. In our definition of this study, fibrinogen levels of < 2 and ≧2 g/L were considered to indicate hypofibrinogen and no hypofibrinogen, respectively.

The study protocol was approved by the Human Ethics Review Committee of the Mie University School of Medicine and a signed consent form was obtained from each subject. This study was faithfully carried out in accordance with the principles of the Declaration of Helsinki.

### The routine assay for the diagnosis of DIC

PT, FDP, fibrinogen and platelet counts were measured as previously reported methods [17, 18].

### The APTT waveform assay

**Fig. 1:**
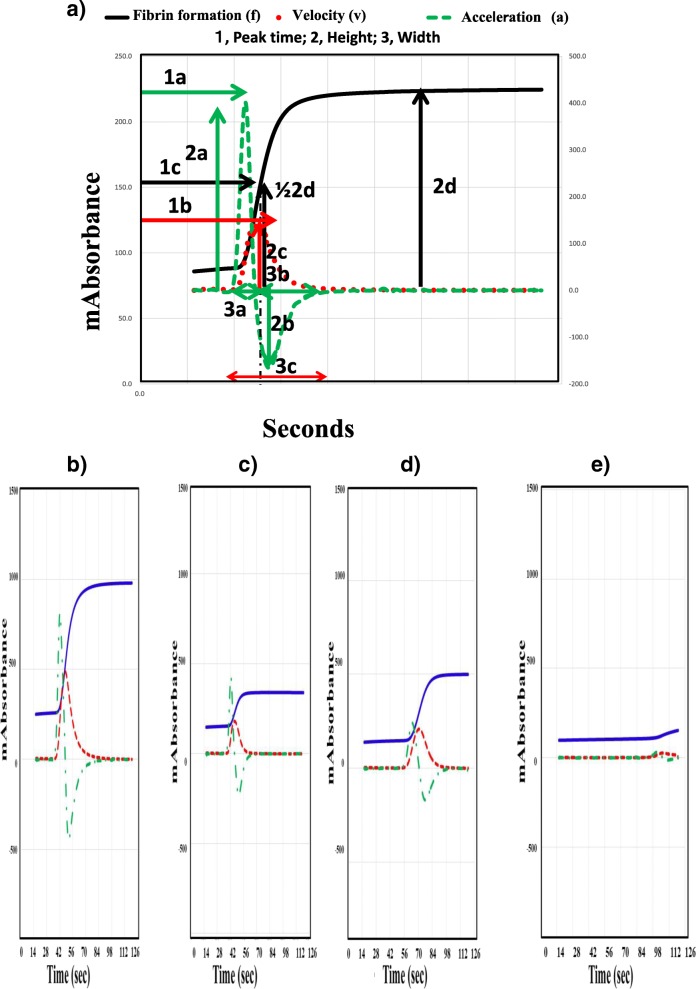
The APTT waveform in a healthy volunteer (**a**), a patient without DIC (**b**), a patient with pre-DIC (**c**), a patients with DIC without hypofibrinogenemia (**d**) and a patient with DIC with hypofibrinogenemia (**e**). a, 2^nd^ derivative; b, negative acceleration, c, 1^st^ derivative; d, coagulation curve, 1, time for peak; 2, height of peak; 3, width of peak

APTT waveform assays were carried out in 30 healthy volunteers (HVs: 10 females and 20 males; median age, 21 years; 25th–75th percentile, 20–24 years) and 211 patients suspected of having DIC. The APTT was measured using the APTT-SP® including silica as an activator of FXII and synthetic phospholipids (Instrumentation Laboratory, Bedford, MA, USA) using an ACL-TOP® system (Instrumentation Laboratory). After measuring the APTT, we performed a waveform analysis based on the results of each APTT assay [15, 19]. Three types of curves are shown on the monitor of the ACL-TOP® system (Fig. 1a); a curve showing the changes in the absorbance observed while measuring the APTT, corresponding to the fibrin formation; a curve showing the first derivative of the absorbance, corresponding to the coagulation velocity; and a curve showing the second derivative of the absorbance, corresponding to the coagulation acceleration. For the waveform analysis, we first checked the presence of an abnormal curve showing a biphasic waveform on the 1^st^DP and/or 2^nd^DP. Furthermore, as shown in Fig. 1a, we calculated the following 10 parameters on the first- or second- derivative curve by manual handling of the mouse: 1, peak time, 1^st^DP, 2^nd^DP1 and 1/2 fibrin formation (1/2FF); 2, width, 1^st^DP, 2^nd^DP1 and 2^nd^DP2; 3, height; 1^st^DP, 2^nd^DP1, 2^nd^DP2 and 1/2FF.

### Statistical analyses

The data are expressed as the medians and 25th–75th percentiles. The differences in factors among HVs, non-DIC patients, patients with pre-DIC and DIC patients with and without hypofibrinogenemia between survivor and non-survivor groups or groups of Hb < 8.0 and ≧8.0 g/L were examined using the Mann-Whitney U-test. A *p*-value of < 0.05 was considered to be statistically significant. All statistical analyses were performed using the Stat Flex, version 6, software package (Artec Co. Ltd., Osaka, Japan).

## Results

The underlying diseases of patients suspected of having DIC was sepsis in 58 (causes for sepsis; pneumonia 30, peritonitis 7, pyelonephritis 5, pleurisy 5, catheter infection 3, necrotizing fasciitis 3, meningitis 2 and others 3), pneumonia in 43, trauma in 41, aneurysm in 15 and hemangioma, living donor liver transplantation in 13, hematopoietic malignancy in 10, cardiopulmonary arrest 10, gynecological complication in 5, autoimmune disease in 4, solid cancer 3 and other infection in 9. Of the 211 patients suspected of having DIC, 65 were diagnosed with DIC (DIC score ≧7), and 25 were diagnosed with pre-DIC (DIC score = 6). Thirty patients showed low levels of fibrinogen (< 2.0 g/L, DIC with hypofibrinogenemia) among 65 patients with DIC (Table 1). Figure 1 shows the APTT waveform in patients without DIC and those with pre-DIC and DIC with and without hypofibrinogenemia. Infectious diseases such as sepsis and pneumonia were 28/35 (80.0%) in DIC without hypofibrinogenemia and 13/30 (43.3%) in DIC with hypofibrinogenemia. The 1stDP and 2ndDP was high in patient without DIC and the width of the 1stDP and 2ndDP were enlarged in patients suspected of having DIC. The height of the 1^st^DP and 2ndDP was reduced in DIC patients, especially in DIC patients with hypofibrinogenemia.

**Table 1:** Non-DIC, Pre-DIC, DIC without hypofibrinogenemia and DIC with hypofibrinogenemia

	Non-DIC	Pre-DIC	DIC without hypofibrinogenemia	DIC with hypofibrinogenemia
Number	121	25	35	30
Age (years)	61 (37–75)	71 (67–80)*	71 (58–76)	63 (54–70)+
Sex (F:M)	60: 61	8: 17	13: 22	13: 17
Platelet counts (10^4^/μl)	15.8 (9.7–26.7)	7.5 (5.0–11.4)***	4.8 (3.3–6.7)***, ^##^	4.4 (2.7–5.9)***, ^###^
PT-ratio	1.1 (1.0–1.2)	1.3 (1.1–1.5)**	1.3 (1.1–1.5)***,	1.4 (1.3–1.7)***, ^#^,^+^
FDP (μg/ml)	13.6 (7.5–29.5)	28.4 (15.0–40.7) **	44.4 (23.2–71.8)***	29.9 (13.8–57.8)***
Fibrinogen (mg/dl)	443 (320–598)	365 (293–449)*	345 (219–404)**,	143 (103–169)***, ^###^, ^+++^
DIC score	3.0 (2.0–4.0)	6.0 (6.0–6.0)***	7.0 (7.0–8.0)***, ^###^	8.0 (7.0–9.0)***, ^###^

*DIC* disseminated intravascular coagulation, *PT* prothrombin time, *FDP* fibrinogen and fibrin degradation products

*, *P* < 0.05; **, *P* < 0.01; ***, *P* < 0.001 compared with non-DIC

^#^, *P* < 0.05; ^##^, *P* < 0.01; ^###^, *P* < 0.001 compared with pre-DIC

^+^, *P* < 0.05; ^+++^, *P* < 0.001 compared with DIC without hypofibrinogen

**Fig. 2:**
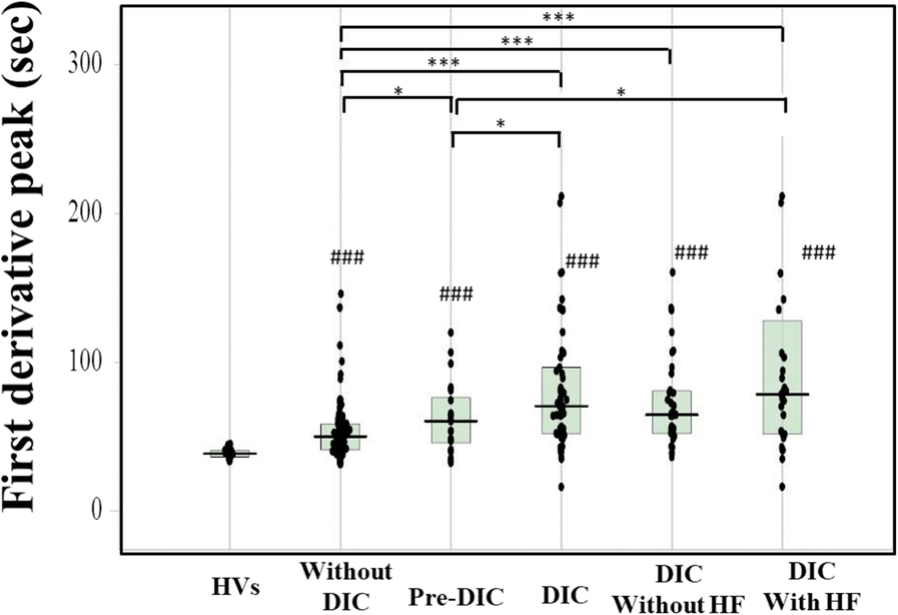
Peak time of 1^st^ derivative (b) of the APTT waveform in healthy volunteers, patients without DIC, patients with pre-DIC, patients with DIC without hypofibrinogenemia and patients with DIC with hypofibrinogenemia. HVs, healthy volunteers; DIC, disseminated intravascular coagulation; HF, hypofibrinogenemia. ###, *p* < 0.001 in comparison with HVs. ***, *p* < 0.001, **, *p* < 0.01; *, *p* < 0.05. HVs, healthy volunteers; DIC, disseminated intravascular coagulation; HF, hypofibrinogenemia. ###, *p* < 0.001 in comparison with HVs. ***, *p* < 0.001, **, *p* < 0.01; *, *p* < 0.05

**Fig. 3:**
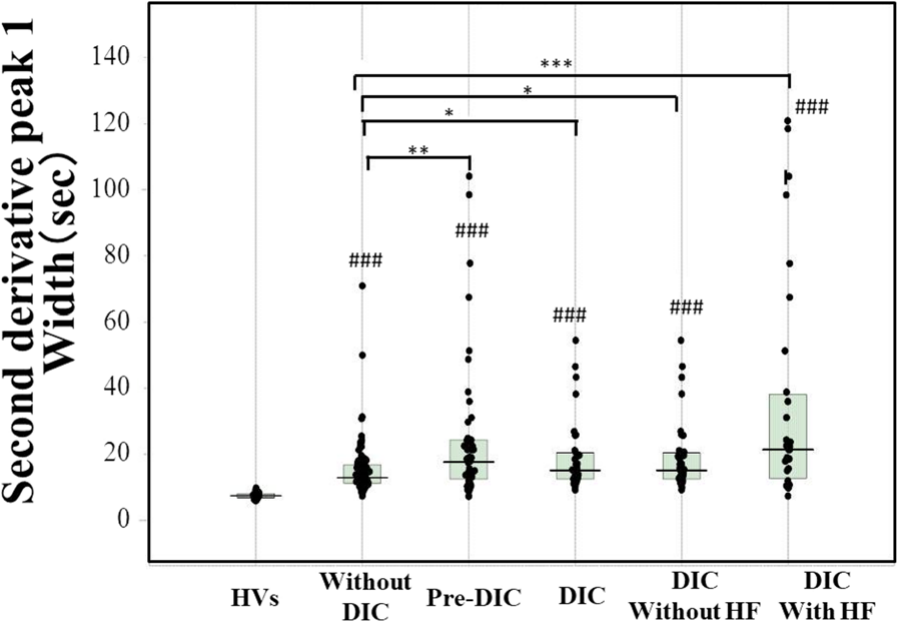
Width of the 2^nd^ derivative-1 of the APTT waveform in healthy volunteers, patients without DIC, patients with pre-DIC, patients with DIC without hypofibrinogenemia and patients with DIC with hypofibrinogenemia. HVs, healthy volunteers; DIC, disseminated intravascular coagulation; HF, hypofibrinogenemia. ###, *p* < 0.001 in comparison with HVs. ***, *p* < 0.001, **, *p* < 0.01; *, *p* < 0.05

**Fig. 4:**
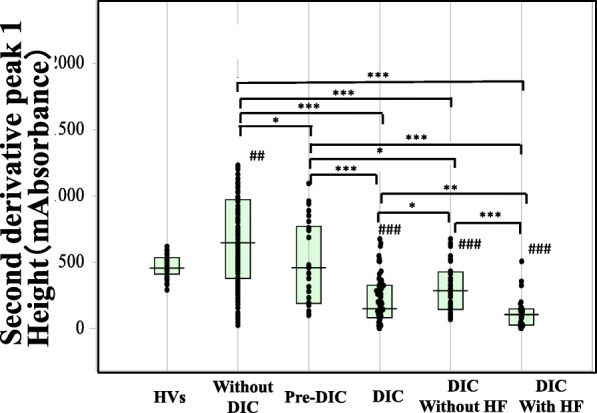
Height of the 2^nd^ derivative-1 of the APTT waveform in healthy volunteers, patients without DIC, patients with pre-DIC, patients with DIC without hypofibrinogenemia and patients with DIC with hypofibrinogenemia. HVs, healthy volunteers; DIC, disseminated intravascular coagulation; HF, hypofibrinogenemia. ###, *p* < 0.001, ##, *p* < 0.01 in comparison with HVs. ***, *p* < 0.001, **, *p* < 0.01; *, *p* < 0.05

**Fig. 5:**
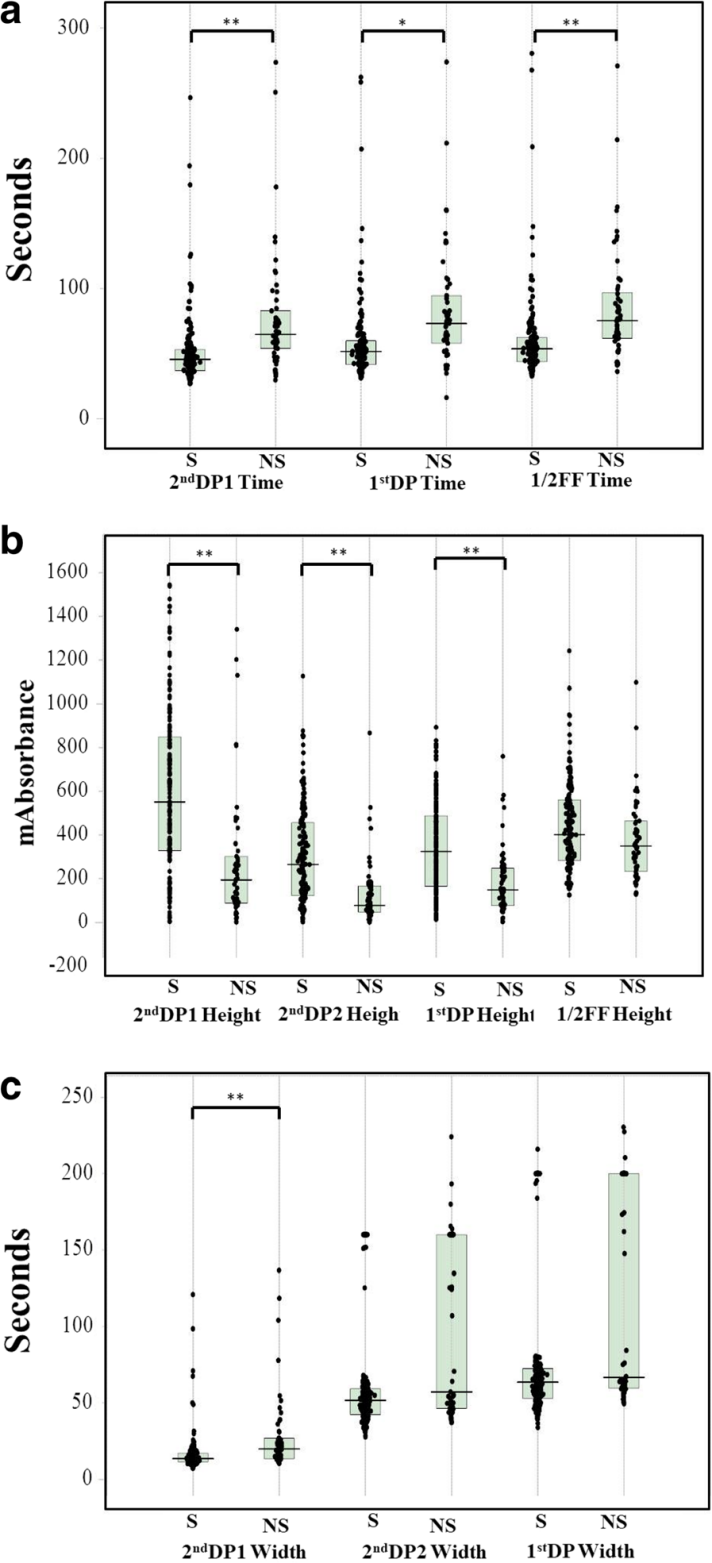
Relationships between the outcome and APTT waveform. Peak time (**a**), peak height (**b**) and peak width (**c**) of the APTT waveform. APTT, activated partial thromboplastine time; DP, derivative peak; FF, fibrin formation; S, survivor; NS, non-survivor. ***, *p* < 0.001, **, *p* < 0.01; *, *p* < 0.05

**Fig. 6:**
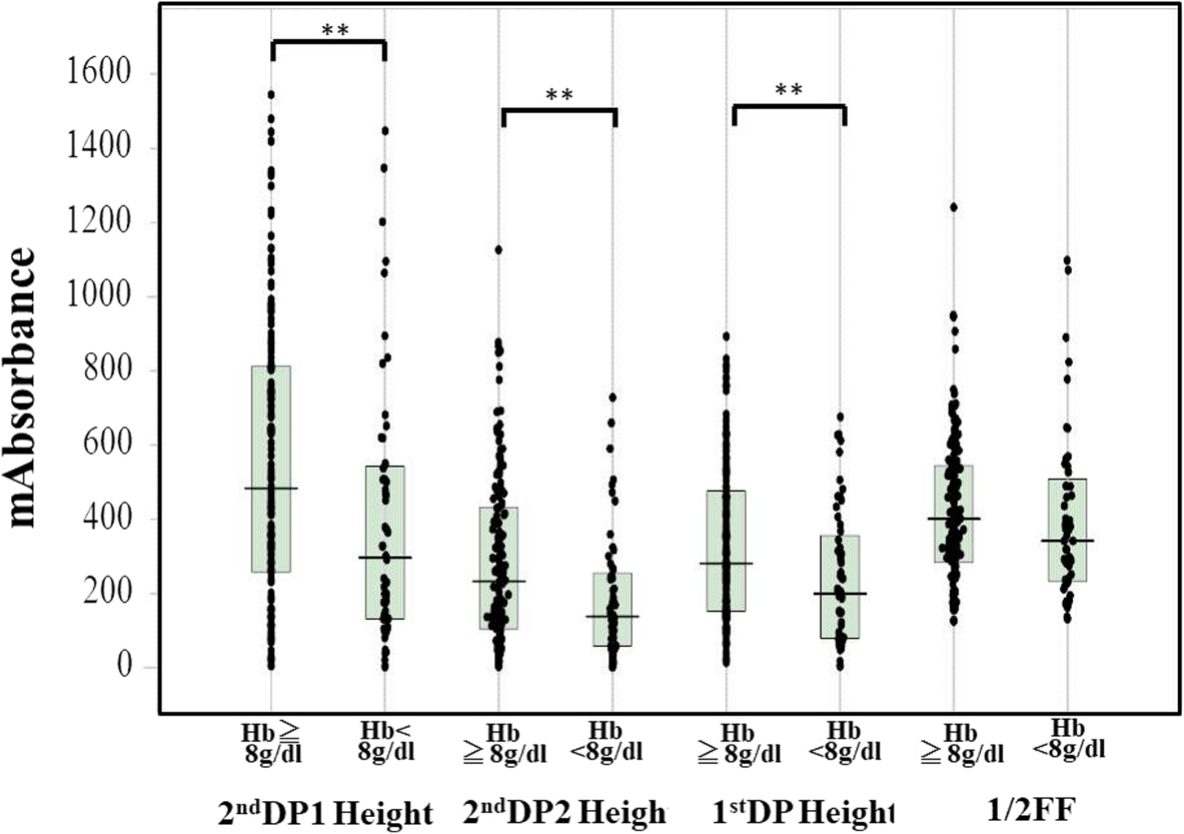
Relationships between hemoglobin level and APTT waveform. The peak height of the APTT waveform. APTT, activated partial thromboplastine time; DP, derivative peak; FF, fibrin formation; S, survivor; NS, non-survivor; Hb, hemoglobin. ***, *p* < 0.001, **, *p* < 0.01; *, *p* < 0.05

The biphasic pattern of 2ndDP was often observed in patients with DIC. The peak time of the 1^st^DP and 2ndDP and 1/2FF were significantly prolonged in patients suspected of having DIC in comparison with HVs (*p* < 0.001) and those were significantly longer in patients with pre-DIC (*p* < 0.05) and in patients with DIC (*p* < 0.001) than in patients without DIC (Fig. 2 and Table 2). There were no significant differences in the peak time of the 1^st^DP and 2ndDP and 1/2FF between DIC patients with and without hypofibrinogenemia. The widths of the 1^st^DP, 2ndDP1 and 2ndDP2 were significantly longer in patients suspected of having DIC (p < 0.001) than in HVs (Fig. 3). The width of the 2^nd^DP1 was larger in patients with pre-DIC (*p* < 0.01) and DIC (*p* < 0.05) than in HVs and that of the 1stDP was longer in patients with DIC than in patients without DIC. There were no significant differences in the 2^nd^DP2 among patients suspected of having DIC. The heights of the 2^nd^DP1 and 2^nd^DP2 were significantly higher in patients without DIC and significantly lower in those with DIC than in HVs (Fig. 4). The height of the 1stDP was significantly higher in patients without DIC and in those with DIC and significantly lower in patients with DIC than in HVs. The height of the 1stDP, 2^nd^DP and 1/2FF were significantly lower (*p* < 0.001) in patients with pre-DIC and those with DIC than in those without DIC. The height of the 1^st^DP, 2^nd^DP1 and 2^nd^DP2 were significantly lower in DIC patients with hypofibrinogenemia than in those without hypofibrinogenemia. The height of the 1^st^DP and 2^nd^DP1 and 2^nd^DP2 were well correlated with DIC score in comparison with the time of the 1^st^DP and 2^nd^DP (Table 3). Fifty five patients died within 28 days form the APTT wave analysis. The time of 2^nd^DP1 (*p* < 0.01), 1^st^DP (*p* < 0.05) and 1/2FF (p < 0.01) was significantly longer in non-survivors (*n* = 55) than in survivors (*n* = 158) (Fig. 5a). The height of 2^nd^DP1 and 2^nd^DP2, and 1^st^DP was significantly lower (*p* < 0.01, respectively) in non-survivors than in survivors (Fig. 5b). The width of the 2^nd^DP1 was significantly larger (*p* < 0.01) in non-survivors than in survivors **(**Fig. 5c). Although the time of the 2^nd^DP was significantly longer (*p* < 0.05) in patients with Hb < 8 g/dl (*n* = 56) than in those with Hb ≧8 g/dl (*n* = 157), there were no significant differences in the time of the 1^st^DP and 1/2FF between patients with Hb ≧8.0 g/dl and those with Hb < 8.0 g/dl **(**Fig. 6). The heights of the 2^nd^DP1, 2^nd^DP2 and 1^st^DP were significantly lower (*p* < 0.01, respectively) in patients with Hb < 8.0 g/dl than in those with Hb ≧8.0 g/dl. The width of the 2^nd^DP1 was significantly larger (*p* < 0.05) in those with Hb < 8.0 g/dl than in those with Hb ≧8.0 g/dl.

**Table 2:** Parameters of APTT waveform in patients with and without DIC and healthy volunteers

		HV	Without DIC	Pre-DIC	DIC	Without HF	With HF
2^nd^DP	Time (seconds)	35.5*** (32.6–37.4)	43.9 (36.6–52.7)	54.6* (41.0–71.3)	63.7*** (47.4–84.9)	58.8*** (46.2–73.9)	72.5*** (49.7–110)
	Height (mabs)	457** (411–535)	648 (379–973))	460* (191–772)	151*** (83.2–326)	286*** (144–427)	107*** (26.5–148)
	NH (mabs)	234** (170–255)	316 (164–499)	176** (93.2–308)	66.4*** (39.4–150)	134*** (61.6–188)	55.0*** (14.5–64.5)
	Width (sec)	7.45*** (6.80–8.00)	12.9 (11.2–16.8)	17.7** (12.5–24.3)	15.1* (12.4–20.5)	15.1* (12.4–20.5)	21.4*** (12.7–38.2)
1^st^DP	Time (sec)	38.7*** (36.3–40.8)	50.2 (41.2–58.6)	60.5* (45.8–76.4)	70.7*** (52.1–96.8)	64.9*** (52.2–81.0)	78.6*** (51.8–128.2)
	Height (mabs)	183*** (144–200))	389 (241–544)	251** (154–358)	103*** (64.2–208)	200*** (116–257)	61.2*** (36.8–93.9)
	Width (sec)	19.5*** (18.0–20.3)	65,2 (54.8–72.1)	65.2 (54.0–195)	66.5 (56.0–200)	64.6 (57.6–200)	75.1 (55.0–200)
1/2FFP	Time (sec)	40.9*** (37.8–42.7)	52.5 (43.1–61.5)	61.6* (47.1–78.9)	75.0*** (54.8–99.3)	69.1*** (54.8–87.9)	80.9*** (54.7–142)
	Height (mabs)	242*** (207–305)	462 (338–586)	328* (271–490)	265*** (178–371)	319*** (240–399)	195*** (170–286)

2^nd^DP, second derivative peak; 1^st^DP, first derivative peak, 1/2FFP, fibrin formation peak; NH, negative height; DIC, disseminated intravascular coagulation; HF, hypofibrinogenemia

Data was shown as median (25–75%tile)

***, *p* < 0.001, **, *p* < 0.01 or: *p < 0.05 compared with patients without DIC

**Table 3:** The Correlation between the DIC score and the parameters of the APTT waveform

Parameters	r	*P*	
2^nd^ derivative peak-1 time	0.2768	*P* < 0.001	Y = 4.74 X + 36.57
2^nd^ derivative peak-1 wide	0.1870	*P* < 0.01	Y = 1.44 X + 12.21
2^nd^ derivative peak-1 height	−0.5342	*P* < 0.001	Y = − 94.56 X + 1002.41
2nd derivative peak-2 wide	0.2991	*P* < 0.001	Y = 6.07 X + 43.55
2nd derivative peak-2 height	−0.5605	*P* < 0.001	Y = −51.87X + 514.24
1^st^ derivative peak time	0.2950	*P* < 0.001	Y = 5.05 X + 40.83
1^st^ derivative peak height	−0.5714	*P* < 0.001	Y = −50.39 X + 553.33
1^st^ derivative peak wide	0.2944	*P* < 0.0001	Y = 7.98 X + 54.30
1/2 fibrin formation time	0.3098	*P* < 0.0001	Y = 5.89 X + 40.64
1/2 fibrin formation height	−0.3593	*P* < 0.0001	Y = −30.37 X + 563.74

*DIC* disseminated intravascular coagulation, *APTT* activated partial thromboplastin time

## Discussion

The APTT waveform analysis indicated that the heights of the 1^st^DP and 2^nd^DP were increased in infectious patient without DIC, and that after the onset of DIC, the widths of the 1^st^DP and 2^nd^DP were enlarged, the heights of the 1^st^DP and 2^nd^DP were reduced, and the biphasic pattern of the 2^nd^DP was often observed. The reduced heights of the 1^st^DP and 2^nd^DP were more significant in patients with DIC with hypofibrinogenemia (bleeding type DIC) than in those with DIC without hypofibrinogenemia. Although septic patients with DIC are usually not associated with bleeding type DIC or hypofibrinogenemia [2, 3], in this study, 43.3% of cases of DIC with fibrinogen level < 2.0 g/L were associated with infectious DIC, indicating that bleeding type DIC with hypofibrinogenemia often occurs in septic patients with DIC. Given that a shift from DIC without hypofibrinogenemia to DIC with hypofibrinogenemia was observed, DIC with hypofibrinogenemia might be a more severe type of DIC than DIC without hypofibrinogenemia. Indeed, the heights of the 1^st^DP, 2^nd^DP1 and 2^nd^DP2 were well correlated with the DIC score, while the heights of the 1^st^DP, 2^nd^DP1 and 2^nd^DP2 were not well correlated with the fibrinogen levels in cases of hemophilia [8].

The peak time of the 1^st^DP and 2^nd^DP and 1/2FF were significantly longer in patients with DIC than in those without DIC, but there were no significant differences in the peak time of the 1^st^DP, 2^nd^DP and 1/2FF between DIC patients with and without hypofibrinogenemia, suggesting that a prolonged peak time of the 1^st^DP and 2^nd^DP and 1/2FF might indicate a diagnostic ability for DIC which is similar to that of a routine APTT assay. A prolonged peak time of the 1^st^DP and 2^nd^DP and 1/2FF are therefore considered to be less useful for the diagnosis of DIC than the height of 1^st^DP and 2^nd^DP. The widths of the 1^st^DP, 2^nd^DP1and 2^nd^DP2 were significant larger in patients suspected of having DIC including both pre-DIC and DIC, than in HVs, suggesting that the width of the APTT waveform indicates the presence of underlying diseases of DIC. The heights of the 2^nd^DP1, 2^nd^DP2 and 1^st^DP were significantly higher in patients without DIC than in those with DIC, suggesting that the heights of the 1^st^DP and 2^nd^DP were markedly high in non-DIC patients suspected of having DIC but reduced in patients with DIC, especially DIC patients with hypofibrinogenemia. As DIC with hypofibrinogenemia is considered bleeding-type DIC, a reduced 1^st^DP and 2^nd^DP might suggest a bleeding risk. The heights of the 2^nd^DP1, 2^nd^DP2 and 1^st^DP were significantly lower in patients with Hb < 8 g/dl than in those with Hb ≧8 g/dl, suggesting that a reducing 1^st^DP and 2^nd^DP might indicate an increased risk for severe bleeding. In orthopedic patients treated with edoxaban, a reduced height of the 1^st^DP was reported to be risk factor for bleeding [15]. Taken together, these findings suggest that anticoagulant therapy should begin in the Pre-DIC state, and strong anticoagulant therapy is not recommended in patients with DIC with hypofibrinogenemia. However, there was no significant difference in the time of the 1^st^DP between the two groups. The height of the 2^nd^DP1 was reported to be well correlated with the FVIII activity [8]. The heights of the 2^nd^DP1, 2^nd^DP2, and 1^st^DP were also observed in bleeding-type DIC. The times of the 2^nd^DP1, 1^st^DP and 1/2FF were significantly longer; the heights of the 2^nd^DP1, 2^nd^DP2 and 1^st^DP were significantly lower; and the width of the 2^nd^DP1 was significantly larger in non-survivors than in survivors. These findings suggest that the APTT waveform might be useful for predicting the outcome.

## Conclusion

As bleeding type DIC was observed in infectious DIC, DIC without hypofibrinogenemia might switch to DIC with hypofibrinogenemia by the progression of DIC. The height of the 1^st^DP and 2^nd^DP is useful for the diagnosis of DIC and prediction of the bleeding risk or outcome in patients with DIC.

## Data Availability

All data are saved in Mie University.

## Abbreviations

1/2FF: 1/2 fibrin formation
aPL: Antiphospholipid antibody
APTT: Activated partial thromboplastin time
DIC: Disseminated intravascular coagulation
DP: Derivative peak
FDP: Fibrinogen and fibrin degradation products
Hb: Haemoglobin
HV: Healthy volunteer
JMHLW: Japanese Ministry of Health, Labor and Welfare
LA: Lupus anticoagulant
PT: Prothrombin time
SP: Synthetic phospholipids
TMA: Thrombotic microangiopathy
UFH: Unfractionated heparin
